# Trapping of Micro- and Nanoparticles within Microfluidic Constrictions in AC Electric Fields

**DOI:** 10.1021/acs.analchem.5c00735

**Published:** 2025-05-22

**Authors:** Raúl Fernández-Mateo, Rahma Gannoun, Hywel Morgan, Antonio Ramos, Pablo García-Sánchez

**Affiliations:** † Depto. Electrónica y Electromagnetismo, Facultad de Física, 16778 Universidad de Sevilla, Avda. Reina Mercedes s/n, 41012 Sevilla, Spain; ‡ School of Electronics and Computer Science, 7423 University of Southampton, Southampton SO17 1BJ, U.K.

## Abstract

Trapping and separation of particles near microfluidic constrictions are efficiently achieved using electric fields. The phenomenon has been attributed to the dielectrophoretic (DEP) force arising from the nonhomogeneous electric field within the constrictions, which predicts particle trapping at or away from the constriction tip. In this work, we provide a more insightful description of the particle behavior around constrictions when subjected to ac electric fields. We demonstrate that, at low frequencies (below 10 kHz) and for solutions with conductivities lower than 0.1 S/m, new trapping positions close to the tips occur which cannot be explained using DEP forces only. We use the term extraordinary trapping position (ETP) to distinguish them from the trapping positions due to DEP. These trapping positions are explained when considering the action of, at least, two different phenomena: the hydrodynamic wall-repulsion induced by concentration-polarization electroosmosis (CPEO) on the particle surface and the fluid flow vortices due to CPEO on the constriction walls. Correctly interpreting these observations is crucial for experiments, such as those aiming to measure the electrical polarizability of proteins by trapping them in microfluidic constrictions.

## Graphical abstract

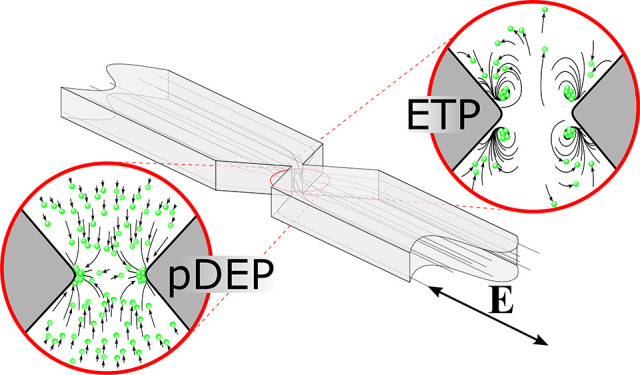

## Introduction

The manipulation of small particles suspended in aqueous solutions can be achieved by applying electric fields.^1,2^ Numerous research groups have demonstrated electric field-induced trapping of particles and molecules within microfluidic channel constrictions.^3^ For instance, early work by Chou et al.^4^ showed that DNA could be trapped and concentrated between insulating obstacles fabricated on a quartz wafer. Cummings and Singh^5^ demonstrated the electrical manipulation and trapping of latex colloids within arrays of glass posts. Lapizco-Encinas et al.^6^ reported the concentration and separation of live and dead bacteria, as well as protein concentration, in low-conductivity electrolytes using DC fields in an array of cylindrical insulating posts etched in glass. Liao et al.^7^ used nanoconstrictions and a combination of AC and DC fields to enrich proteins in physiological media. Also, Physher and Hayes used a series of constrictions with decreasing width along a channel and demonstrated the separation of bacterial populations after application of a DC field.^8^

A common feature in all the experiments mentioned above is the use of electric fields applied across constrictions and/or obstacles within a channel. The electric current is forced through narrow gaps, generating a spatially nonuniform electric field. When a polarizable particle is introduced into this nonuniform field, it experiences a net electrical force, leading to particle movement known as dielectrophoresis (DEP).^9,10^ When this field distortion is caused by insulating structures within the channels, such as pillars or constrictions, the method of particle manipulation is referred to as insulating DEP (iDEP) or electrodeless DEP (eDEP), though the term eDEP is more commonly associated with electrode-based DEP.

In a recent work,^11^ we demonstrated that the DEP force does not provide a complete picture of the behavior of micro- and nanoparticles subjected to AC electric fields within constrictions. Instead, we experimentally showed that steady-state quadrupolar flow vortices around the constrictions dominate the particle motion at low frequencies (<10 kHz), which were successfully described by the novel theory of Concentration Polarization Electroosmosis (CPEO). This model provided a framework for describing time-averaged electrosmostic flows induced around charged insulating structures such as insulating micropillars^12,13^ and microspheres.^14^ Briefly, CPEO describes an electroosmotic flow caused by the variation in electrolyte concentration around charged objects driven by surface conductance upon application of an external electric field. This means that the time scale is dominated by diffusion, and thus CPEO vanishes for frequencies above the reciprocal of the diffusion time (2π*f* > *D*/*a* ^2^, where *D* is the diffusion constant and *a* a typical length), where DEP is observed.

The new picture in our previous work^11^ combined the action of CPEO and DEP: CPEO causes particles to trace loops around the constrictions, and DEP makes particles accumulate either at the tip of the constrictions (positive DEP or pDEP) or away from it (negative DEP or nDEP) depending on particle size and liquid conductivity. Interestingly, we also reported an accumulation of particles at intermediate positions on both sides of the constriction for specific configurations of particle size, channel heights and frequencies of the ac field. We referred to that accumulation (see Figure 5b of that work, reproduced here in Figure 2) as *trapping*, which is notably different from either nDEP or pDEP accumulation positions (Figure 2 of ref 11). Similar behavior was also found around insulating pillars (18 μm diameter) subjected to ac fields in a shallow channel (8 μm depth).^15^

Subsequent research^16^ has revealed that the electric fields acting on charged dielectric particles induce a hydrodynamic repulsion from the microchannel walls. The origin of this interaction is the CPEO flows induced on the particle surface. Notably, this hydrodynamic repulsion exceeds the repulsion caused by the electrostatic interaction between the induced dipole on the particle and its image dipole in the wall. This new understanding of wall-particle interactions motivated us to revisit the unexplained trapping observed in our previous experiments. The main goal of this work is the study of this particle accumulation at intermediate frequencies of applied electric fields. To this end, we performed new experiments and characterized the particle accumulation, which we refer to as Extraordinary Trapping Positions (ETP), as a function of electrolyte conductivity, channel height, particle size and particle charge sign. More importantly, we performed numerical simulations to calculate particle trajectories, explicitly accounting for the hydrodynamic repulsion revealed in ref 16. We demonstrate that ETP arises from a combination of, at least, two different phenomena: the hydrodynamic wall-repulsion induced by CPEO on the particle surface and the CPEO vortices on the constriction walls. The simulations also include electrophoresis and dielectrophoresis, and they predict trapping regardless of the sign of the particle charge. It is also found that ETP does not occur for particles larger than a maximum size *a* because nDEP dominates, in accordance with experimental results.

The paper is organized as follows: First, we show the results of the experimental characterization of the ETP. Second, we describe the physical mechanisms that we consider in the simulations of the particle trajectories and within the microfluidic constrictions. Finally, we compare experiments with the simulations of the trajectories. The results of this comparison clarify much of the interplay between different ac electrokinetic forces that determine the particle behavior in applications such as iDEP devices or Electrokinetic Deterministic Lateral Displacement (DLD) devices.^15,17–20^

## Experiments

### Experimental Setup and Methods

Microfluidic devices were fabricated in PDMS using standard soft lithography techniques. We fabricated wide channels (200 μm) with a constriction at the center, measuring 20 μm in width (Figure 1). The channels were produced with two different heights: 50 μm (referred to as tall channels) and 10 μm (shallow channels), which are typically used in iDEP experiments.^4,5^ They were filled with aqueous KCl solutions with conductivities of σ = {1.7, 6.1, 12.2} mS/m and a pH of approximately 5.5. Polystyrene fluorescent particles of various sizes and zeta potentials (see Table 1) were dispersed in the solutions. The particles were imaged using a microscope with a × 100 objective. Before filling the channels, they were primed with a 0.1% (w/v) Pluronic F-127 solution, a nonionic surfactant that adsorbs onto the channel wall surface, reducing particle adhesion.

**Table 1:** Size and Zeta Potential of Particles Used in the Work

diameter	functionalization	zeta potential (mV)
2 μm	carboxyl	–72 ± 7
3 μm	carboxyl	–81 ± 8
500 nm	amidine	56 ± 6
1 μm	amidine	49 ± 5

**Figure 1:**
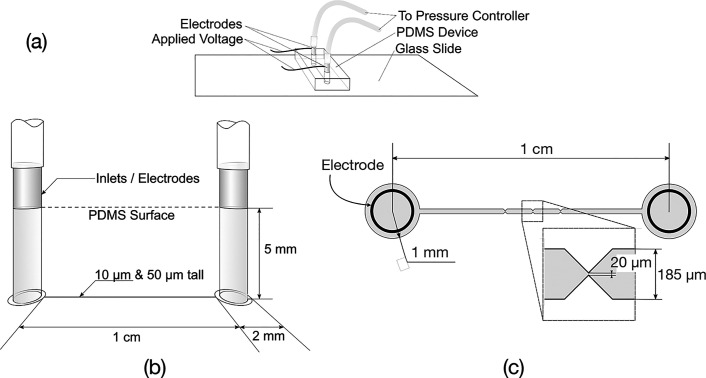
Diagrams of the experimental setup. (a) Overview of the device with connections to the voltage source to generate the electric field, and the pressure regulators controlling the fluid flow. (b) Microfluidic device showing electrode connections that also serve as fluid inlets. (c) Detailed top view of the channel and constriction showing dimensions.

The electric field was generated using two metal needles, positioned at the inlet and outlet of the channel, with a 1 cm gap between them. One needle was grounded, while the other was connected to alternating current (AC) voltages with peak-to-peak amplitudes reaching up to 1600 V. A pressure controller (Elveflow OB1MK3+) was used to replenish the liquid in the channel and halt the flow between measurements. This liquid refreshment helps reduce variations in electrical properties caused by Faradaic reactions, which occur due to the low-frequency electric field.

### Experimental Results

We first verified the repeatability of the experimental observations from our previously published work.^11^ The new results with 500 nm and 1 μm particles are in complete agreement with the earlier findings, which are reproduced in Figures 2 and 3. Figure 2 shows a map describing the behavior of 500 nm diameter particles around microfluidic constrictions in shallow channels (10 μm). These particles were dispersed in a KCl aqueous solution with conductivity 1.7 mS/m. The colors on the map represent different regions in the amplitude-frequency space where distinct regimes were observed: at low frequencies of the ac electric field (*f* ≤ 100 Hz), the amplitude of electrophoretic motion is large and the particles move from one side of the constriction to the other. For frequencies higher than 100 Hz, the amplitude of the electrophoretic oscillations vanish and, depending on the amplitude of the applied voltage, we either observed the quadrupolar flows caused by CPEO or ETP of the 500 nm particles. The positions of particle trapping are shown in the experimental image inserted in the figure. If the frequency is increased above 10 kHz, the particles also become trapped within the microfluidic constriction but at a different position – the particles are trapped at the tip of the constriction. This attraction to the tips is consistent with the behavior of particles undergoing positive DEP, as expected for particles having this size in a liquid with a conductivity of 1.7 mS/m. However, the trapping shown in Figure 2 cannot be accounted for DEP forces and, importantly, it was not observed with 500 nm particles in tall channels. It is important to emphasize that these phenomena are not necessarily mutually exclusive, and in some regions of the map, multiple effects coexist. To distinguish these, we used solid symbols to mark points where a single behavior clearly dominates, while open symbols indicate regions where multiple behaviors overlap.

**Figure 2:**
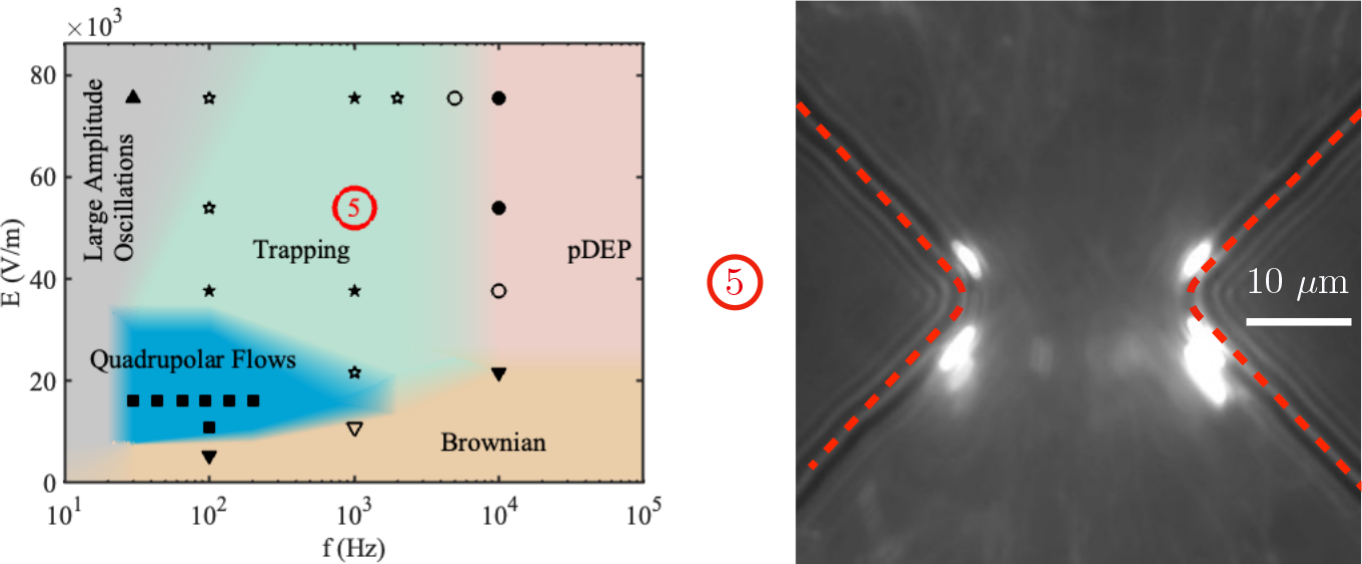
Voltage versus frequency map describing the regimes observed around a shallow constriction (10 μm tall). The particles are 500 nm diameter dispersed in a KCl solution with conductivity 1.7 mS/m. The colors on the map represent different regions in the amplitude-frequency space where distinct behavioral regimes were observed. Solid symbols indicate points where a single behavior clearly dominates, while open symbols indicate regions where multiple behaviors overlap. Reprinted with permission from ref 11. Copyright 2021, American Chemical Society.

**Figure 3:**
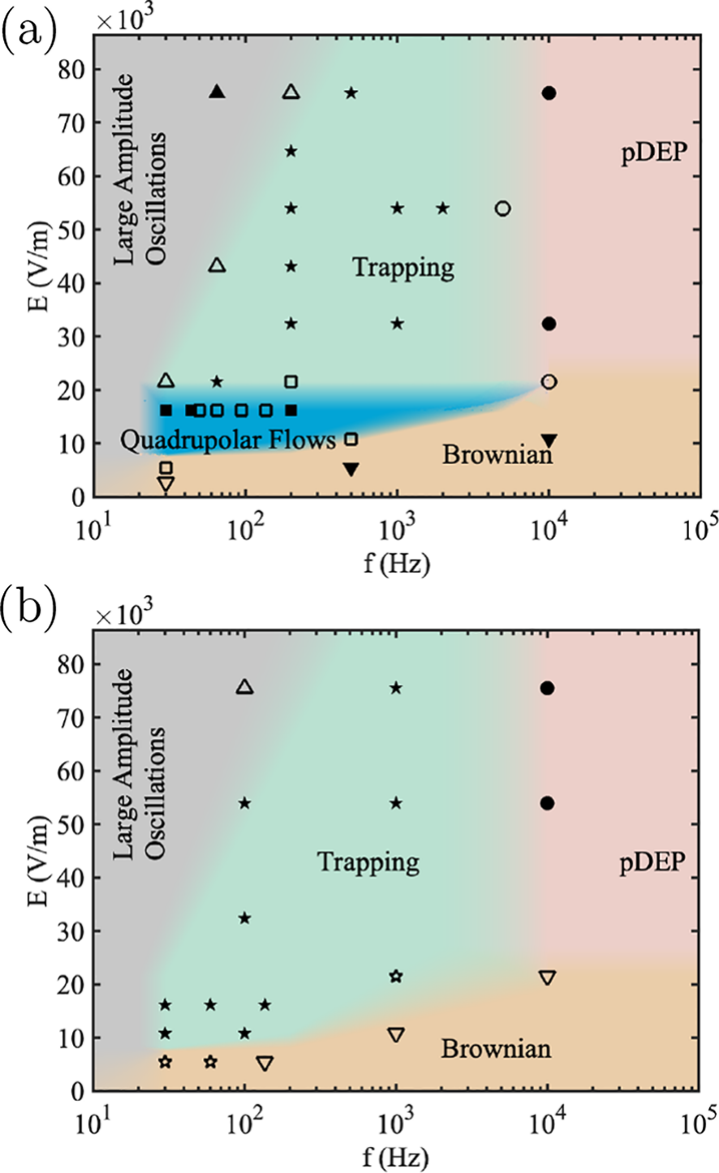
Voltage versus frequency map describing the regimes observed with 1 μm diameter particles dispersed in a KCl solution with conductivity 1.7 mS/m. (a) Tall channels (50 μm) and (b) shallow channels (10 μm). The colors on the map represent different regions in the amplitude–frequency space where distinct behavioral regimes were observed. Solid symbols indicate points where a single behavior clearly dominates, while open symbols indicate regions where multiple behaviors overlap. Reprinted with permission from ref 11. Copyright 2021, American Chemical Society.

While ETP did not occur for 500 nm particles in tall channels, it was easily observed for 1 μm particles in both tall and shallow channels. This is shown in Figure 3a, where either quadrupolar flows or ETP are observed depending on the frequency of the applied voltage. Also, Figure 3b describes the observations with 1 μm diameter particles in shallow channels. In this case, we did not observe the quadrupolar flow rolls.

We carried out further experimental work to determine the characteristics of the particle ETP. Figure 4a,b show maps describing the behavior of 2 μm diameter particles dispersed in a KCl solution with conductivity 1.7 mS/m. For this case, ETP has been found for intermediate frequencies for both tall and shallow channels. However, when the conductivity of the liquid was increased to 6.1 mS/m, the ETP regime was not observed and the 2 μm particles moved away from the constrictions, as expected if they experienced negative DEP. Further experiments with 3 μm diameter particles were performed and the ETP regime was not observed regardless of the solution conductivity where these particles were dispersed.

**Figure 4:**
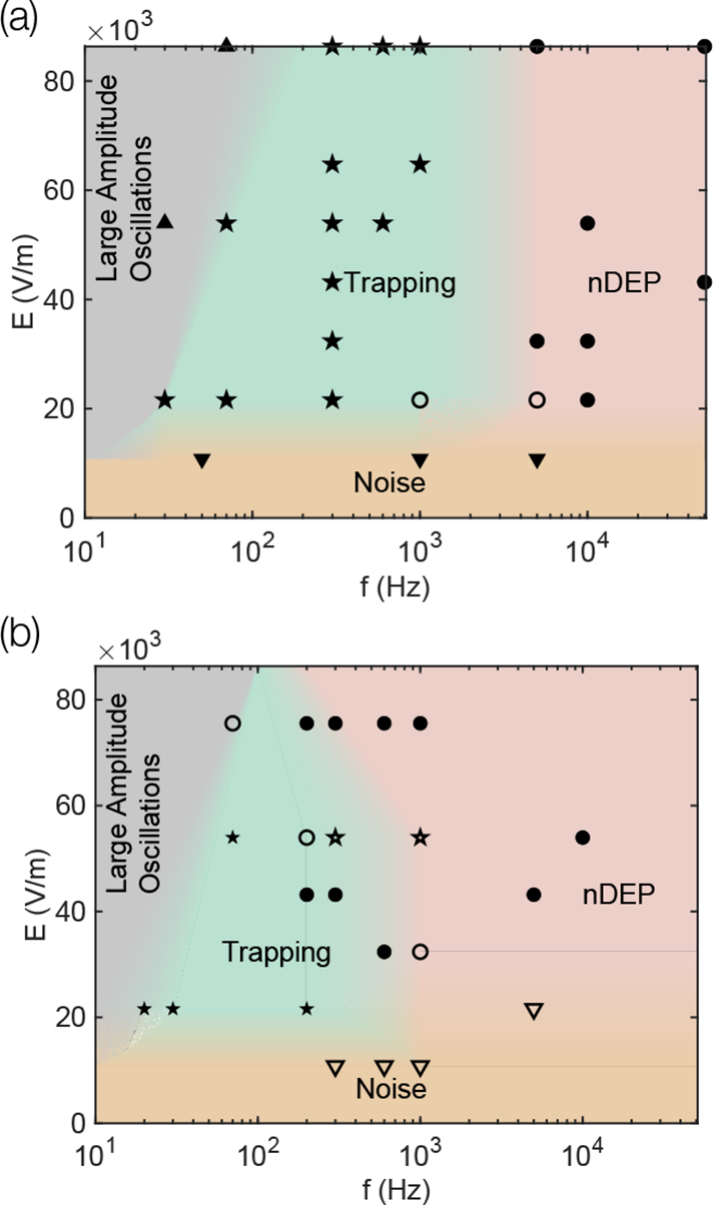
Voltage versus frequency map describing the regimes observed with 2 μm diameter particles dispersed in a KCl solution with conductivity 1.7 mS/m. (a) Tall channels (50 μm) and (b) shallow channels (10 μm). The colors on the map represent different regions in the amplitude-frequency space where distinct behavioral regimes were observed. Solid symbols indicate points where a single behavior clearly dominates, while open symbols indicate regions where multiple behaviors overlap.

All previous experiments have been performed with carboxylated polystyrene particles, which bear a negative surface charge. In order to check the possible influence of the sign of the surface charge, we also carried out experiments with polystyrene particles coated with amidine, which gives the particles a positive surface charge. We used positive charged particles of 500 nm and 1 μm diameter (see zeta-potential in Table 1). Experiments with the amidine-coated particles showed the same behavior as the carboxylated particles (maps in Figures 2 and 3) and thus we ruled out the sign of the zeta-potential as a significant parameter in the appearance of the ETP regime.

In light of the experimental observations described above, key conditions for the appearance of ETP are

- **AC electric fields of intermediate frequencies**. If the frequency is too low (<100 Hz), oscillatory electrophoresis dominates the particle motion. If the frequency is high (>1 kHz), no flow rolls are observed and DEP governs the particle motion. Extraordinary Trapping was found for typical frequencies where vortices due to CPEO are observed. That is, the electric field frequency must be of the order or lower than the inverse of the time for ionic diffusion across the typical length of the constriction, *f* ∼ *D*/(2π*d* ^2^), *d* the distance between the constriction tips.^11^ This also limits the conductivity of the electrolytes, σ ≪ 100 mS/m.^11^
- **Particle size**. Experiments show that the particle size must be smaller than a critical value, depending on the liquid conductivity. In particular, no Extraordinary Trapping was observed for 2*a* ≳ 3 μm (*a* is the particle radius).
- **Extraordinary Trapping is independent of the sign of the particle charge**. While the use of AC voltages in the experiments might suggest that the sign of the particle charge has no effect, the theory of concentration polarization predicts the formation of a DC electric field near the constriction due to surface conduction, potentially driving net electrophoretic motion. Nevertheless, experimental results unambiguously showed that ETP occurred for both positively and negatively charged particles.

## Theory and Numerical Simulations

In order to accurately describe the particle motion at the constrictions, we calculated numerically particle trajectories using the software COMSOL Multiphysics complemented with MATLAB including the following contributions to the particle velocity.

### Electrophoresis

Electrophoresis refers to the motion of charged particles suspended in an electrolyte subjected to an electric field. The particle motion arises from the action of the electric field on the charges within the electrical double layer (EDL).^21^ When the EDL is much thinner than the particle size, the electrophoretic velocity of the particle **u**_ep_ is given by the Helmholtz-Smoluchowski formula,^22^

$$u_{\mathrm{ep}}=\frac{\varepsilon \zeta }{\eta }E$$ 1

where ε and η are the electrolyte permittivity and viscosity respectively, and ζ is the zeta potential, which is usually defined as the electrical potential at the inner edge of the diffuse ionic layer surrounding the particle.^21^ The applied electric field **E** is numerically computed for the specific geometry of the constriction as will be shown later in this section.

### Dielectrophoresis

The application of an electric field along a constriction gives rise to a spatially nonuniform electric field. In this situation, a net electrical force is exerted on a suspended polarizable particle and the resulting particle motion is known as dielectrophoresis (DEP).^9,10^ Considering a small particle, i.e. particle size smaller than the typical length scale for variation of the electric field, the DEP force can be calculated from the expression of the force acting on the electrical dipole induced on the particle (**F** = (**p** ·∇)**E**, where **p** is the induced dipole moment). For the case of a spherical particle of radius *a*, subjected to an ac field of angular frequency ω and given by $E=E_{0}Re\left[\exp\left(i\omega t\right)\right]$ , the time-averaged DEP force acting on the particle can be written as

$$F_{\mathrm{DEP}}=\pi \varepsilon a^{3}Re\left[f_{\mathrm{CM}}\left(\omega \right)\right]\nabla |E_{0}{|}^{2}$$ 2

where *f*_CM_(ω) is the frequency-dependent nondimensional particle polarizability, commonly known as the Clausius-Mossotti factor.^9^ When *f*_CM_(ω) > 0, the DEP force points toward the region of higher electric field – positive DEP (pDEP). Otherwise, if *f*_CM_(ω) < 0 the force points toward lower electric fields – negative DEP (nDEP).

For small Reynolds numbers, the viscous drag on a spherical particle of radius *a* moving with velocity **u** within a fluid with viscosity η is written as **F**_drag_ = 6πη*a* **u**. Thus, the contribution to the particle velocity due to DEP is

$$u_{\mathrm{DEP}}=\frac{\varepsilon a^{2}}{6\eta }Re\left[f_{\mathrm{CM}}\left(\omega \right)\right]\nabla |E_{0}{|}^{2}$$ 3

### Particle Repulsion from the Microchannel Walls

Another consequence of applying an electric field to the particles is that they are repelled from the channel walls. In principle, this repulsion has two distinct origins: First, the induced electrical dipole in the particle interacts with its image dipole due to the presence of a channel boundary leading to a dipole–dipole repulsion away from the wall. Second, stationary quadrupolar flows are induced around charged particles suspended in low conductivity electrolytes (≲0.1 S/m) under the action of ac electric fields^14^ as shown in Figure 5a. We recently showed that such flows lead to particle repulsion from nearby walls.^16^ For low frequencies of the ac fields (<10 kHz), this repulsion is much stronger than dipole–dipole interaction.^23^ The quadrupolar flows are caused by Concentration-Polarization Electroosmosis (CPEO).^13^ i.e. electroosmosis arising from gradients in electrolyte concentration around the particles.

**Figure 5:**
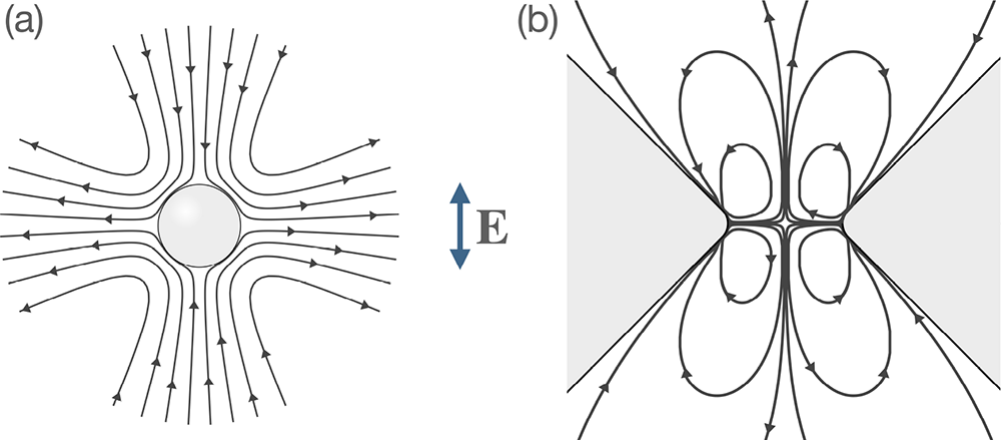
Streamlines of the fluid velocity field predicted by CPEO in an AC electric along the vertical direction. (a) Sphere. (b) Microfluidic constriction.

According to Figure 5a, the quadrupolar flow induced on the particle surface has a symmetry given by the direction of the electric field. As a consequence, the hydrodynamic interaction with a nearby wall depends on the direction of the electric field with respect to the wall. This interaction can be either attractive, repulsive or parallel to the wall.^24^ In our case, the electric field will be primarily parallel to the channel walls and, therefore, the interaction will be mainly repulsive.^16^ If the distance of the particle center to the wall is *h*, the repulsion velocity far from the wall (*a*/*h* ≪ 1) can be calculated using the reflection of its CPEO flow,^25^ and may be expressed as^24,26^

$$u_{\mathrm{rep}}=v_{0}\frac{3a^{2}}{8h^{2}}ẑ$$ 4

where *ẑ* is a unit vector perpendicular to the wall. The parameter *v*_0_ is the CPEO maximum slip velocity at the surface of the particle, which can be determined analytically using the CPEO model^14^ or indirectly through measurements of wall repulsion of particles undergoing electrophoresis.^23^

### CPEO Vortices Induced on the Microchannel Walls

CPEO vortices occur on charged insulating surfaces such as the surface of the particles and the channel walls.^13^ In a recent work,^11^ we reported stationary vortices around constrictions as drawn in Figure 5b, which were successfully described according to the theory of CPEO. The effect of these CPEO flows is also included in the simulation of particle trajectories. As will be shown later, it turns out that the effect of these vortices on the particle velocity is critical for the description of trapping.

In order to simulate the CPEO vortices, we follow the small Dukhin analysis of the model presented in a previous work.^13^ The Dukhin number Du is defined as the ratio of surface to bulk conductance,^27^ Du = *K*_s_/σ*d*, with *K*_s_ the surface conductance, σ the electrolyte conductivity and *d* a characteristic geometrical length scale. In the present problem, we chose *K*_s_ = 1 nS as a typical value for charged colloids,^28^ σ ∼ 5 mS/m and *d* = 20 μm is the constriction width. This results in Du ∼ 10^–2^, which falls within the limits of the approximation.

This limit allows linearizing the electric potential ϕ and the electrolyte ion concentration *c* with the Dukhin number: ϕ = ϕ_0_ + δϕ and *c* = *c*_0_ + δ*c*, respectively. That is, we write the magnitudes as the sum of a contribution disregarding surface conductance (Du = 0) plus a perturbed contribution due to its small effects, which we denote with “δ”. The linearization of the electrokinetic equations in the case of ac voltages gives rise to a steady-state velocity field that scales linearly with Du but is quadratic with the amplitude of the electric field, with a time-averaged slip velocity at the wall surface given by

$$\left\langle u_{\mathrm{slip}}\rangle =\frac{\varepsilon \zeta_{0}}{\eta }\langle \nabla_{s}\delta \phi \rangle -\frac{\varepsilon \phi_{\mathrm{ther}}}{\eta c_{0}}\tanh\;\frac{\zeta_{0}}{2\phi_{\mathrm{ther}}}\langle \delta c\nabla_{s}\phi_{0}\right\rangle$$ 5

where ζ_0_ is the zeta-potential of the surface ignoring surface conduction effects, *c*_0_ is the bulk ion concentration and the thermal voltage (ϕ_ther_ = *k*_B_ *T*/*e* ≈ 25 mV) is used as the scale for electric potential. The remaining equations and boundary conditions for the electric potential, ion concentration and fluid velocity field are presented in Figure 6. We refer the reader to our previous publications on CPEO for a complete derivation and detailed interpretation.^13,14^ Crucially, the boundary condition for δ*c* on the channel walls accounts for surface conduction, leading to concentration polarization (CP).

**Figure 6:**
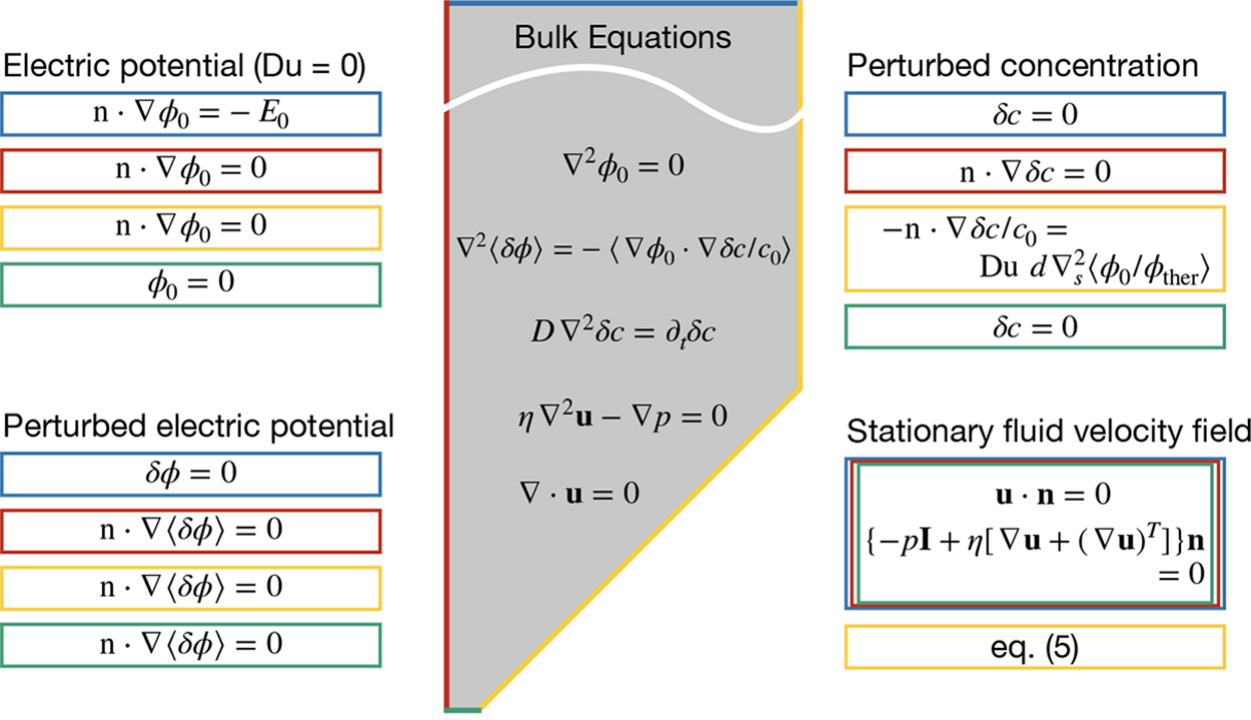
Domain (gray shaded area), bulk equations and boundary conditions for computing the CPEO flows around the constriction following the small Dukhin number analysis. The domain is chosen to utilize the symmetry of the geometry, meaning only 1/4 of the constriction is meshed. Boundary conditions for the four computed fields are matched with their corresponding boundary using same colors. In the equations, **n** stands for the unit vector normal to the wall and *p* is the pressure field.

The electric field used to compute Electrophoresis and DEP, along with the CPEO flows described above (Figure 6) were obtained with finite element method simulations using COMSOL Multiphysics. The domain was meshed using methods suggested by the software with additional refinement to the corner boundary where CP occurs (maximum element size equal to 1% of the constriction gap and 5 boundary layers). The resulting fields were then exported to MATLAB as nodes, and particle trajectories were computed with time steps containing at least 25 points per AC period. Intermediate positions of trajectories between exported nodes where linearly interpolated.

### Results of the Numerical Simulations

The electric field in the microchannel which was obtained to calculate the CPEO flows (see Figure 6) was used to calculate the other contributions to the particle velocity, i.e., the electrophoresis, dielectrophoresis and the particle-wall interaction, resulting in a total particle velocity **u**_total_ given by

$$u_{\mathrm{total}}=u_{\mathrm{ep}}+u_{\mathrm{DEP}}+u_{\mathrm{rep}}+v\left(r\right)$$ 6

where the last contribution, **v**(**r**), is the CPEO fluid velocity evaluated at the particle position, which is induced on the channel walls. Thus, these velocity calculations can include the effects of surface conductance in the evaluation of the electric field, increasing the accuracy of the particle trajectories. Also, given that the electric field is not completely parallel to the constriction walls, the hydrodynamic particle-wall interaction will have attractive and parallel components with respect to the walls.^20,24^ However, we checked that these contribution are negligible compared with the repulsive interaction given that the perpendicular component of the electric field to the wall is negligible when compared with the parallel components. Therefore, we omit the other terms and compute only the repulsion velocity **u**_rep_ induced by the parallel component of the electric field to the wall at the particle position.

First, simulations are validated against previous published results. Specifically, we simulate 500 nm beads (ζ_p_ = – 60 mV) suspended in a 1.7 mS/m and 12.2 mS/m KCl electrolyte, in a triangular constriction of 20 μm gap with a zeta potential of ζ = – 25 mV (PDMS treated with surfactant, as explained in the Experiments section). This behavior matches the experimental trajectories in our previous experiments^11^ (see maps of Figure 2 in that publication). Figure 7 shows the particle trajectories computed using MATLAB as described above. Each subfigure contains 100 particle trajectories starting from random initial positions and spanning a fixed amount of time which gives an idea of the relative velocity at different positions, and also between subfigures (Figure 7d contains an extra 20 trajectories due to extremely low velocities).

**Figure 7:**
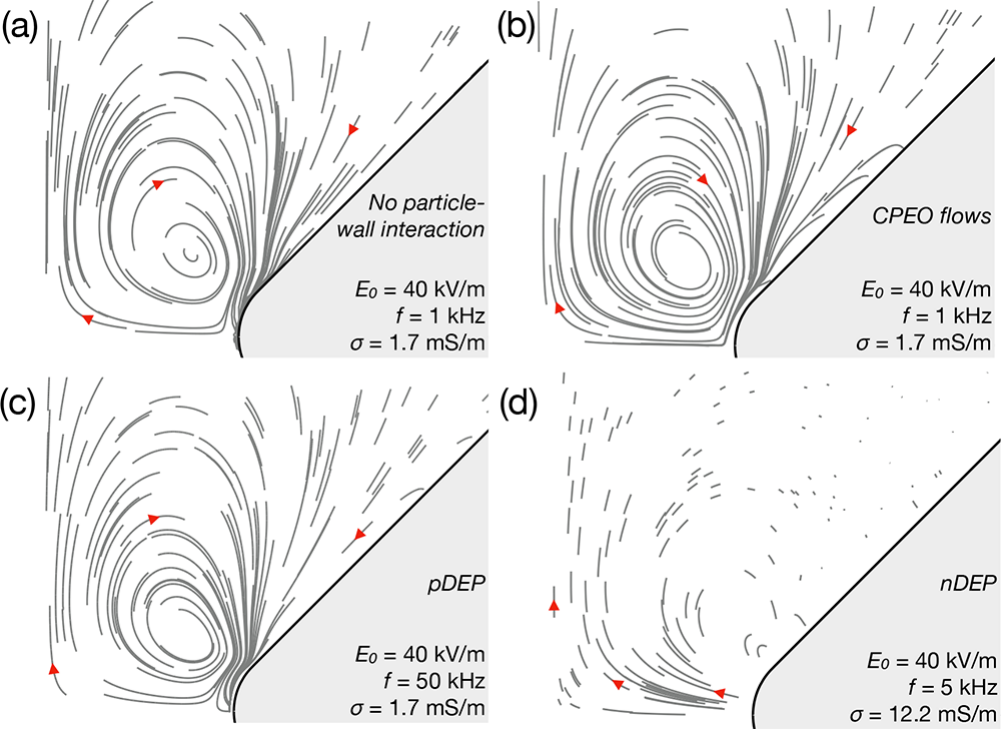
Simulations of 500 nm particle trajectories in the microfluidic constriction for different electric fields and conductivity regimes to reproduce previous experimental observations.^11^ (a) and (b) Trajectories for 40 kV/m and 1 kHz: while the simulation in (a) does not include the wall repulsion, the simulation in (b) includes the particle-wall repulsion due to CPEO flows. (c) Trajectories for an electric field of 40 kV/m and 50 kHz. The particles experience pDEP at this frequency. (d) Trajectories for an electric field of 40 kV/m and 5 kHz. The liquid conductivity is 12.2 mS/m and the particles experience nDEP. Each subfigure has *N* = 100 particle trajectories starting from random positions and with a fixed time span of *t* = 0.05*t*_0_ = 0.05η*d* ^2^/(εϕ_ther_^2^) = 0.045 s. Red arrows mark the orientation of the trajectories. Trajectories are averaged over one period *T* = 1/*f* for visualization.

Figure 7a,b shows the same electric field configuration: 40 kV/m magnitude and 1 kHz frequency in a σ = 1.7 mS/m conductivity electrolyte. Subfigure (a) does not include contributions from the particle-wall hydrodynamic force. The particles follow the CPEO vortices and accumulate at the tip of the constrictions due to pDEP. However, the trajectories shown in subfigure (b) do include the effect of wall repulsion and no accumulation at the tip constriction is observed. In other words, the wall repulsion due to CPEO around particles prevents trapping due to pDEP.

To observe pDEP phenomena in simulations, in subfigure (c) we increased the frequency to 50 kHz without changing the remaining parameters. It shows how the particles near the constriction are trapped at the tips. Finally, increasing the conductivity will eventually lead to nDEP. In addition, this will reduce the magnitude of the CPEO flows which decay with increasing conductivities (both of the constrictions and around particles causing particle-wall repulsion), making DEP observable at lower frequencies. In Figure 7d we reduce the electric field frequency to 5 kHz and increase the conductivity to 12.2 mS/m. This shows how particle velocities are extremely slow as they get farther from the tip due to weak CPEO effects. Near the constriction tip, we observe how particles are rapidly expelled from the walls due to nDEP as previously seen in experiments.

Remarkably, the wall repulsion mechanism is also necessary to understand the Extraordinary Trapping reported in our previous work. In Figure 8a, we show trajectories of 1 μm particles (ζ = – 70 mV) under the action of CPEO flows around the constriction and DEP when subjected to an electric field of 40 kV/m and a frequency of 1 kHz in an electrolyte conductivity of 1.7 mS/m. In this case, pDEP dominates and particles accumulate at the constriction tip. However, if wall repulsion is considered in the simulations, as shown in Figure 8b, particles are attracted to a specific loop independently of their initial position around the constriction in less than *t*_0_ = η*d* ^2^/(εϕ_ther_^2^) ≈ 0.9 s. These loops were reported as trapping positions in our previous publication.^11^ Simulations combining the three forces acting on the particles (i.e., CPEO flows, wall repulsion and DEP) show ETP depends on the relative dominance of wall repulsion over pDEP. If DEP is omitted from the simulation in the same conditions, as presented in Figure 8c, the final trapping loops are more confined to specific positions and thus are made more apparent.

**Figure 8:**
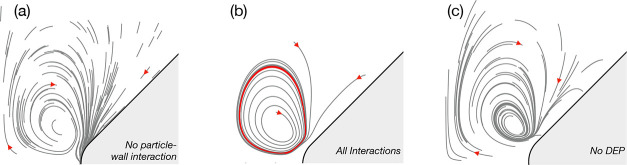
Extraordinary trapping predicted by the simulations for 1 μm particles. (a) Accumulation at the constriction tip as predicted by DEP if no particle-wall interaction is considered. 100 particles are simulated for a fixed time of *t* = 0.05*t*_0_. (b) ETP arises if wall-repulsion is introduced in the simulations, and particles starting from different positions are rapidly (*T* < *t*_0_ ≈ 0.9 s) attracted to a single loop, whose size varies depending on particle properties. Three trajectories are computed for *t* = *t*_0_, and the last 20% of trajectories time are drawn in red to show the final common loop. (c) If DEP is ignored to compute the trajectories, trapping is magnified to more confined loops. 50 particles are simulated for a fixed time of *t* = 0.1*t*_0_. For all simulations, we used *E*_0_ = 40 kV/m, *f* = 1 kHz, σ = 1.7 mS/m.

This observation helps understand the conditions for the appearance of ETP: a strong wall repulsion combined with a weak DEP action will maximize the effect. Thus, particles with high surface conductance (resulting in a strong wall-particle interaction) but with conductivity similar to that of the suspending medium (which will minimize the effects of DEP) should demonstrate strong trapping. It turns out that particle size is a critical parameter in the appearance of ETP. We can use eqs 3–5, validated through comparison between simulations and experiments, to obtain scaling laws for particle size using the parameters shown in Figure 9 (σ = 1.7 mS/m, *E*_0_ = 50 kV/m, *f* = 1 kHz, ζ = – 70 mV). Resulting curves are shown in Figure 9a by comparing the velocity of the CPEO flows around the constrictions *v* and the velocity resulting from DEP force on particles *u*_DEP_ with the velocity of wall-particle repulsion *u*_rep_, i.e. *v*/*u*_rep_ and *u*_DEP_/*u*_rep_:

$$\frac{u_{\mathrm{DEP}}}{u_{\mathrm{rep}}}∼\frac{4}{9}\frac{\varepsilon a^{2}V^{2}}{\eta d^{3}v_{0}}Re\left[f_{\mathrm{CM}}\right],\frac{v}{u_{\mathrm{rep}}}∼\frac{8v}{3v_{0}}$$ 7

where ∇|**E**_0_|^2^ ∼ *V* ^2^/*d* ^3^, *h* ∼ *a*, and *v*, *v*_0_ are computed using the expressions from literature.^13,14^ Additionally, background colors in Figure 9a indicate the dominant regime as a function of particle size obtained after complete numerical simulations using the same parameters (see Figure caption). Figure 9a confirms that we expect ETP for intermediate particle sizes, not too small to follow CPEO vortices and not high enough for strong nDEP.

**Figure 9:**
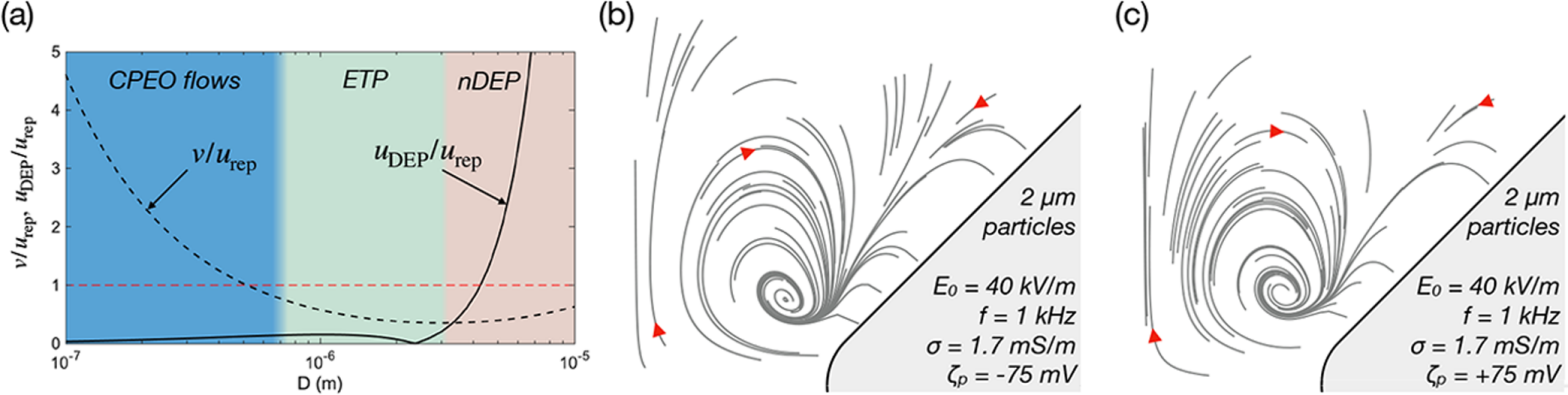
Extraordinary Trapping arises in the combination of strong hydrodynamic particle-wall interaction and weak DEP force. (a) Plot of the CM factor for the usual experimental conditions (*E*_0_ = 40 kV/m, *f* = 1 kHz, σ = 1.7 mS/m, ζ_p_ = – 70 mV). The colored regions correspond to results of numerical calculations of particle trajectories. Extraordinary Trapping is confirmed when trajectories collapse into a single loop independently of their initial position in the region of interest (ROI, see for example subfigure (b)) within *t* = *t*_0_. (b) Averaged trajectories of 2 μm particles in the same experimental conditions and ζ_p_ = – 75 mV using a time span of 0.1*t*_0_, where a clear ETP is observed. (c) shows same numerical conditions but inverting the particle zeta potential sign.

Calculations predict that a particle of 2 μm will experience strong trapping as observed in the trajectories plotted in Figure 9b. In this scenario the trapping loop is visibly reduced to a point for simulation times of *t* = 0.1*t*_0_. According to the CPEO theory,^13,14^ ETP is unaffected by the sign of the zeta potential of the particle. In Figure 9c we present the same conditions as in subfigure (b) but reversing the sign of the particle zeta potential, demonstrating no significant change between both calculations.

As expected by the simulations, CPEO flows around the constriction occur for small particle sizes given that they become tracers of the fluid flow. On the other hand, large particles are expelled from the constriction tips as the (negative) DEP force dominates given the strong dependence on particle size. It is only in the region where neither fluid flows nor DEP dominates that ETP arises, influenced by the presence of CPEO-driven wall repulsion.

Finally, we note that Brownian motion was not included in the simulations, as our primary objective was to capture ETP resulting from the interplay between DEP, CPEO-induced vortices, and wall repulsion. Nonetheless, particles, especially nanoparticles, are subject to Brownian motion. This effect is clearly observed in experiments conducted with low electric field amplitudes. To address this, simulation were performed for 500 nm particles at different electric field strengths (see Supporting Information). The results demonstrate that, at low field amplitudes, Brownian motion dominates over CPEO flow. In contrast, simulations for 1 and 2 μm diameter particles confirm that Brownian motion has a negligible impact on ETP.

## Conclusions

In this work we have studied the particle accumulation recently observed around microfluidic constrictions both theoretically and experimentally. To identify this novel phenomenon we use the term Extraordinary Trapping Position (ETP). Experimentally ETP was observed below 10 kHz in solutions with conductivities lower than 0.1 S/m. Using the theory of CPEO and the particle-wall hydrodynamic interaction it induces on charged dielectric particles, we were able to explain previous experimental observations, and predict new configurations for the appearance of this phenomenon. This shows the relevance and power of CPEO in understanding the behavior of dielectric particles in microfluidics.

However, there remain behaviors that cannot be explained using the combination of forces presented in this work, with current approximations. For example, all three interactions considered in this work (CPEO, wall repulsion and DEP) scale equally with the electric field magnitude squared, so that it remains unclear why the change between quadrupolar flows and ETP regimes varies with *E*_0_, as shown in some experimental maps. Although this transition is not always present, there are scenarios in which this was clear. This could be attributed to deviations from the quadratic response of the CPEO,^23^ which is expected to play a more significant role in the quadrupolar flow that occurs at the constrictions, reducing the velocity compared to predictions in the low-electric-field model. Consequently, this alters the force balance in the system.

## Supplementary Material



## Notes

Detailed description of the simulations of particle trajectories including the effects of Brownian motion (PDF)
