# Supplementary material for: Trapping of Micro- and Nanoparticles within Microfluidic Constrictions in AC Electric Fields
Source: Anal Chem. 2025 May 22;97(21):11165–73. doi: 10.1021/acs.analchem.5c00735 (PMC12138874; doi:10.1021/acs.analchem.5c00735)
Supplement: Supplementary file 1 [file ac5c00735_si_001.pdf]

SUPPORTING INFORMATION

Trapping of micro- and nano-particles within microfluidic constrictions in AC electric fields

Raúl Fernández-Mateo[1], Rahma Gannoun[1], Hywel Morgan[2], Antonio Ramos[1], and Pablo García-Sánchez[1]

[1]School of Electronics and Computer Science, University of Southampton, Southampton SO17 1BJ, United Kingdom.

[2]Depto. Electrónica y Electromagnetismo. Facultad de Física. Universidad de Sevilla. Avda. Reina Mercedes s/n, 41012. Sevilla, Spain.

Contents:

S1 Influence of Brownian motion on the simulations of particle trajectories

S1 Influence of Brownian motion on the simulations of particle trajectories

The simulations of particle trajectories shown in the main text do not include stochastic motion therefore we have included Brownian motion into the calculations to evaluate the effects. This is performed using the Langevin equation, which governs the dynamics of a particle under thermal fluctuations along with deterministic forces. The stochastic force F_{rand} is modeled as a Gaussian noise term with a variance determined by the fluctuation-dissipation theorem as $\langle F_{\text{rand}}^2 \rangle = 2k_B T\gamma/\Delta t$, where $\gamma = 6\pi\eta a$ is the Stokes particle drag coefficient. In this equation, k_B is the Boltzmann constant, T the absolute temperature, and Δt the time step of the simulations.

This force can be rewritten in terms of particle diffusion coefficient D_p which is related to temperature and drag through the Einstein relation, $D_p = k_B T/\gamma$,

$$\mathbf{F}_{\text{rand}} = \gamma\sqrt{\frac{2D_p}{\Delta t}}\mathbf{W}, \tag{1}$$

where $\mathbf{W}$ is a vector whose components are random numbers sampled from a Gaussian distribution. To implement this in the simulations, in each time step we introduce a displacement term $\delta\mathbf{r}_{\text{rand}} = \mathbf{W}\sqrt{2D_p\Delta t}$:

$$\mathbf{r}(\Delta t) = \mathbf{r}_0 + \frac{\mathbf{F}}{\gamma}\Delta t + \delta\mathbf{r}_{\text{rand}}, \tag{2}$$

where $\mathbf{F}$ is the deterministic force applied on the particle.

Validation of the transition from the Brownian motion regime to CPEO flow

First, we checked that the Brownian terms incorporated in the equations correctly described the observed particle behaviour. This should reproduce the observed transitions between, for example, CPEO flows and Brownian motion as described in our previous work [1]. Figure S1 shows traces of 500 nm particles due to CPEO flows at 40 kV/m applied electric field amplitude (a) to trajectories dominated by Brownian motion at 5 kV/m (d). All cases were calculated for a field frequency of 1 kHz and 1.7 mS/m KCl electrolyte conductivity, reproducing the conditions used in Figure 2(a) of Ref. [1]. Panels (a) and (b) show that particle motion is dominated by CPEO, panel (c) shows a combination of the two phenomena while finally panel (d) shows that Brownian motion dominates making it impossible to observe CPEO flows over a typical observation timescale. This is agreement with experimental observations presented in our previous work [1].

Influence of Brownian motion on ETP

Having validated the addition of the Brownian component to particle trajectories with experiments, its effect was included in conditions where ETP is expected, i.e. for particle sizes of 1 μm and 2 μm in diameter as shown in the main text.

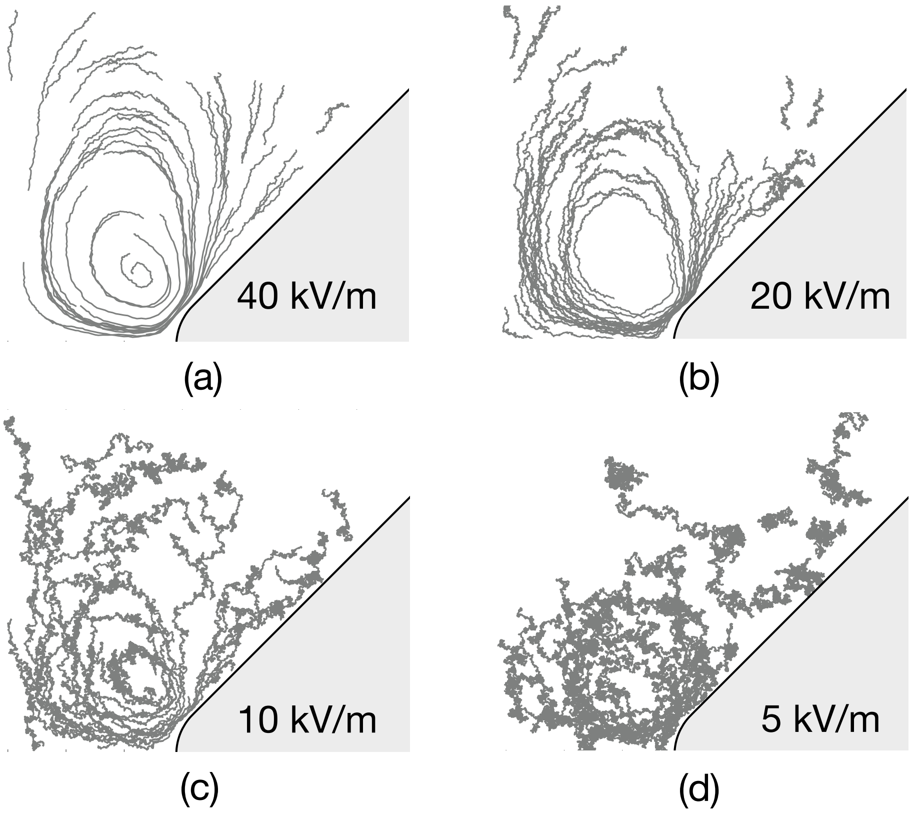

Figure S1: Simulation of particle trajectories replicating Fig. 2(a) of Ref. [1]: 500 nm particles suspended in 1.7 mS/m KCl electrolyte. Electric field amplitude varies from (a) 40 kV/m to (d) 5 kV/m with 1 kHz ac frequency. Figure shows how Brownian motion distorts the CPEO flows traced by the particles during the observed time windows.

Figure S2 shows trajectories of particle undergoing ETP. Panel (a) is for 1 μm particles with the same conditions as in Figure 8(b) of the main manuscript (40 kV/m electric field amplitude, 1 kHz ac frequency, and 1.7 mS/m electrolyte conductivity). Fig. S2(b) is for the same conditions but including Brownian motion., demonstrating that this does not prevent particles from being trapped in the recirculating loops. Similar results are found for the larger 2 μm particles, in other words Brownian motion does not have a significant effect on ETP.

As observed in Figure S2, the effects of Brownian motion for the 2 μm particles is barely noticeable, consistent with the particle diffusion coefficient scaling inversely with particle size.

References

[1] Raúl Fernández-Mateo, Víctor Calero, Hywel Morgan, Antonio Ramos, and Pablo García-Sánchez. Concentration–polarization electroosmosis near insulating constrictions within microfluidic channels. *Analytical Chemistry*, 93(44):14667–14674, 2021.

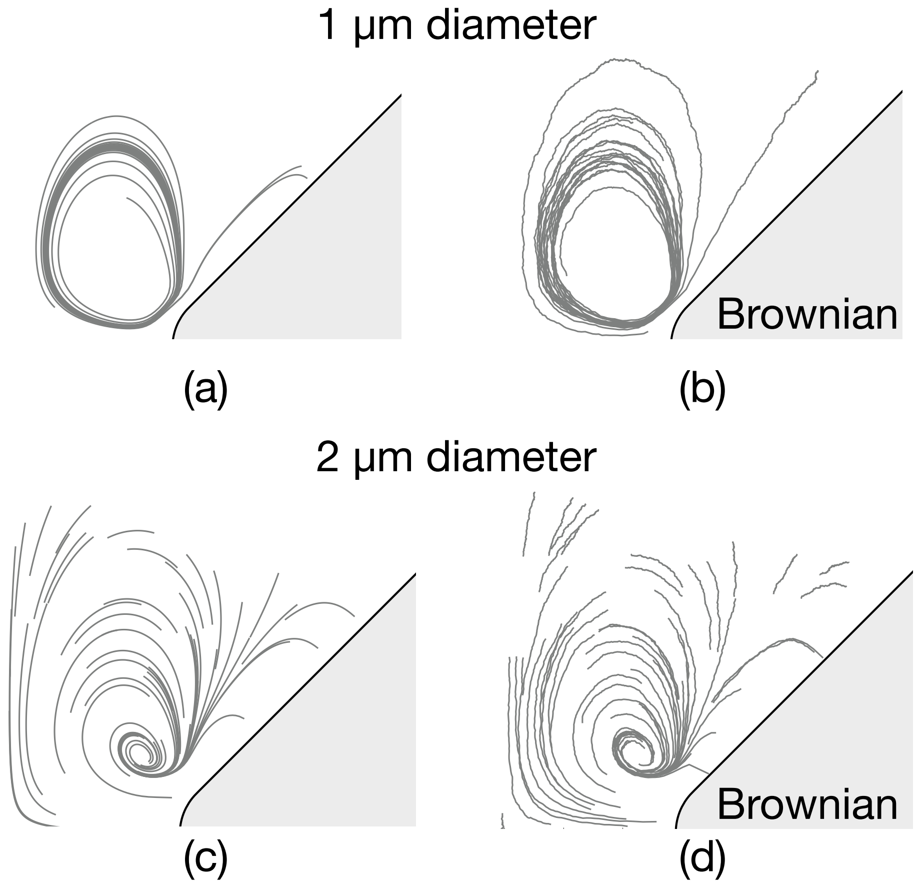

Figure S2: Comparison of particle without Brownian motion (a) and (c), and including Brownian motion (b) and (d). Particle sizes are 1 μm (top row) and 2 μm (bottom row) in diameter. Electric field is 40 kV/m amplitude, 1 kHz frequency and a 1.7 mS/m KCl electrolyte. The images show that Brownian motion does not have a significant effect on ETP.
